# Neural control of the circulation during exercise in health and disease

**DOI:** 10.3389/fphys.2013.00224

**Published:** 2013-08-26

**Authors:** Paul J. Fadel

**Affiliations:** Department of Medical Pharmacology and Physiology, Dalton Cardiovascular Research Center, University of MissouriColumbia, MO, USA

**Keywords:** exercise pressor reflex, arterial baroreflex, blood pressure, sympathetic nerve activity, vascular responses to exercise

During exercise, appropriate cardiovascular, and hemodynamic adjustments are necessary to meet the metabolic demands of active skeletal muscle. Autonomic alterations in sympathetic and parasympathetic nerve activity play a major role in ensuring these adjustments are adequately made. Several neural mechanisms working in concert are responsible for regulating this autonomic activity and through complex interactions, control the cardiovascular and hemodynamic changes in an intensity-dependent manner. Central command (a feed-forward mechanism originating from higher brain centers), the exercise pressor reflex (EPR; a feed-back mechanism originating in skeletal muscle), the arterial baroreflex (a negative feed-back mechanism originating from the carotid sinus and aortic arch), and the cardiopulmonary baroreflex (a negative feed-back mechanism originating from the heart, and blood vessels of the lungs) are all known to contribute to the neural cardiovascular adjustments to physical activity. This research topic was designed to provide an update in the area of neural control of the circulation and present some of the newest work and current ideas in this continually progressing field. Indeed, fundamental mechanisms driving each of these neural inputs continue to be elucidated. Moreover, there is a growing interest in regard to alterations in the activity of each input after the development of cardiovascular disease. The papers compiled in this e-book highlight some of the most recent work regarding neural cardiovascular control during exercise in health and disease using a variety of experimental models and methodologies.

Given its importance in mediating the cardiovascular responses to exercise, the EPR (activated via excitation of group III and IV afferent fibers within skeletal muscle), has been a major focus of past and current research. This is highlighted by 4 contributions in this research topic. An important advancement is the growing understanding of impairments in EPR function accompanying the development of cardiovascular disease. Indeed, sympathetic activation during exercise is exaggerated in a number of disease states increasing the risk of a cardio- and/or cerebral-vascular event while also contributing to exercise intolerance. For example, EPR dysfunction has been shown to contribute to the augmented exercise-induced sympatho-excitation in heart failure and hypertension. In this research topic, for the first time, alterations in muscle temperature are considered as a possible contributing factor to the exaggerated EPR activity in heart failure (Li et al., 2012). More importantly, Li and Xing (2012) extend the list of diseases with impairments in EPR function to include peripheral arterial disease. These authors summarize recent mechanistic work demonstrating a role for the EPR in contributing to the augmented sympathetic and pressor responses to exercise in peripheral arterial disease. With the goal of treating EPR dysfunction, Wang et al. (2012) provide a thorough review of the mechanisms that contribute to the exaggerated EPR in heart failure, while demonstrating the effectiveness of chronic exercise training as a non-pharmacological therapy to ameliorate heightened EPR-induced sympathetic activation. This is accompanied by a paper by Mueller and Mischel (2012) in which regular physical activity is shown to modulate excitatory/inhibitory neurotransmission within the rostral ventrolateral medulla, a brainstem region integral in the central control of sympathetic outflow. Thus, exercise training can have important beneficial central as well as peripheral effects that modulate sympathetic control of the circulation. Lastly, Murphy et al. (2013) provide novel insight into the timing at which EPR function may be altered following injury to the spinal cord; information critical to the implementation of exercise prescription in patients with spinal cord injury (a population known to be at an increased risk for the development of cardiovascular disease).

Another reflex critical for appropriate neural cardiovascular adjustments to exercise is the arterial baroreflex. It is well established that the arterial baroreflex resets to remain functional during exercise and plays an important role in ensuring appropriate neural cardiovascular responses are elicited. Notably, the majority of work contributing to our knowledge of the arterial baroreflex in humans during exercise has been performed in young Caucasian men. In this research topic, Holwerda et al. (2013) have begun to extend these findings to young African Americans clearly showing for the first time a similar magnitude of resetting in this group compared to young Caucasian Americans. However, impairments in the ability of African Americans to defend against a hypertensive challenge were observed during steady-state exercise, providing novel information that may begin to explain the greater cardiovascular responsiveness to physiological stressors in African Americans compared to Caucasian Americans. Another interesting concept put forth in this research topic is provided by Schwartz and Stewart (2012). As noted above, although arterial baroreflex resetting is well known to occur during exercise, less information is available regarding other forms of stress. These authors provide data suggesting the presence of resetting during upright tilt with the sympathetic baroreflex being augmented and potentially contributing to an increase in peripheral resistance that may improve an individual's ability to defend against hypotension. This is important information that highlights the need for additional human studies to further characterize resetting during orthostasis.

While reflex control of sympathetic outflow is critical, vascular changes ultimately determine the pressor responses evoked. Indeed, augmented central sympathetic activation would be minimized by insufficient transduction into a vascular response. Thus, vascular responses are critical to fully elucidating the neural control of the circulation during exercise. Importantly, sympathetic responses are only one facet of vascular control competing with metabolic, hormonal, mechanical, shear-induced, conducted, myogenic, and autoregulatory mechanisms. In this research topic, Matsukawa et al. (2013) provide a mini-review on vasodilator responses at the onset of exercise and the potential contribution of central command in mediating a cholinergic and/or beta-adrenergic vasodilator signal to skeletal muscle. Casey and Joyner (2012) examine the interaction between sympathetically-mediated vasoconstriction and vasodilation in the partial restoration of flow following hypoperfusion evoked by intra-arterial balloon inflation. They demonstrate that alpha-adrenergic mediated vasoconstriction restricts compensatory vasodilation during forearm exercise with hypoperfusion, but is not responsible for the initial increase in vascular resistance at the onset of hypoperfusion. These findings further underscore the interactions and complexities of vascular control during exercise. Interestingly, this is likely extended to post exercise as well. Indeed, Moynes et al. (2013) demonstrate a persistent attenuation of vascular responses to sympathetic activation acutely after the cessation of exercise that may have important implications for blood pressure regulation and immediate post exercise hypotension. Lastly, it is important to remember that not all vascular beds respond alike. For the most part, skeletal muscle blood flow has been discussed; however, increases in skin blood flow as well as cerebral blood flow are also important in the adjustments to exercise. Miyazawa et al. (2012) demonstrate that the regulation of these vascular beds are likewise quite complex.

Overall, this research topic highlights recent advances in the neural control of the circulation in health and disease and at the same time, continues to demonstrate the diversity and complexities involved. Indeed, the more we learn, the more we appreciate the integration needed to coordinate appropriate neural cardiovascular and hemodynamic adjustments to exercise as well as the plasticity of the neural cardiovascular system in both health and disease. We now await further advances in this important field of research.
